# A Multi-Analyte Approach for Improved Sensitivity of Liquid Biopsies in Prostate Cancer

**DOI:** 10.3390/cancers12082247

**Published:** 2020-08-11

**Authors:** Lilli Hofmann, Katja Sallinger, Christoph Haudum, Maria Smolle, Ellen Heitzer, Tina Moser, Michael Novy, Kevin Gesson, Thomas Kroneis, Thomas Bauernhofer, Amin El-Heliebi

**Affiliations:** 1 Division of Cell Biology, Histology and Embryology, Gottfried Schatz Research Center, Medical University of Graz, 8010 Graz, Austria; lilli.hofmann@medunigraz.at (L.H.); katja.sallinger@medunigraz.at (K.S.); thomas.kroneis@medunigraz.at (T.K.); 2 Center for Biomarker Research in Medicine (CBmed), 8010 Graz, Austria; christoph.haudum@medunigraz.at; 3 Division of Endocrinology and Diabetology, Department of Internal Medicine, Medical University of Graz, 8010 Graz, Austria; 4 Department of Orthopaedics and Trauma, Medical University of Graz, 8010 Graz, Austria; maria.smolle@medunigraz.at; 5 Institute of Human Genetics, Diagnostic and Research Center for Molecular BioMedicine, Medical University of Graz, 8010 Graz, Austria; ellen.heitzer@medunigraz.at (E.H.); tina.moser@medunigraz.at (T.M.); 6 BioTechMed-Graz, 8010 Graz, Austria; 7 Christian Doppler Laboratory for Liquid Biopsies for Early Detection of Cancer, Medical University of Graz, 8010 Graz, Austria; 8 ViennaLab Diagnostics, 1120 Vienna, Austria; novy@viennalab.com (M.N.); gesson@viennalab.com (K.G.); 9 Division of Oncology, Department of Internal Medicine, Medical University of Graz, 8010 Graz, Austria; thomas.bauernhofer@medunigraz.at

**Keywords:** CTCs, prostate cancer, AR-V7, in situ padlock probe, *AR* amplification, liquid biopsy, multi-analyte

## Abstract

Novel androgen receptor (AR) signaling inhibitors have improved the treatment of castration-resistant prostate cancer (CRPC). Nonetheless, the effect of these drugs is often time-limited and eventually most patients become resistant due to various AR alterations. Although liquid biopsy approaches are powerful tools for early detection of such therapy resistances, most assays investigate only a single resistance mechanism. In combination with the typically low abundance of circulating biomarkers, liquid biopsy assays are therefore informative only in a subset of patients. In this pilot study, we aimed to increase overall sensitivity for tumor-related information by combining three liquid biopsy approaches into a multi-analyte approach. In a cohort of 19 CRPC patients, we (1) enumerated and characterized circulating tumor cells (CTCs) by mRNA-based in situ padlock probe analysis, (2) used RT-qPCR to detect cancer-associated transcripts (e.g., *AR* and *AR*-splice variant 7) in lysed whole blood, and (3) conducted shallow whole-genome plasma sequencing to detect *AR* amplification. Although 44–53% of patient samples were informative for each assay, a combination of all three approaches led to improved diagnostic sensitivity, providing tumor-related information in 89% of patients. Additionally, distinct resistance mechanisms co-occurred in two patients, further reinforcing the implementation of multi-analyte liquid biopsy approaches.

## 1. Introduction

Prostate cancer (PC) is one of the most common malignant cancers in men causing more than 350,000 deaths worldwide per year [1]. As PC is linked to a dysregulation of the androgen receptor (AR) signaling pathways, the most common treatment involves androgen deprivation therapy (ADT). Although the majority of patients benefit from ADT, patients become resistant at a high rate, resulting in castration-resistant PC (CRPC) [2]. At this stage, novel AR (androgen receptor) signaling inhibitors (ARSI; e.g., enzalutamide and abiraterone) represent effective therapeutic options for 60–80% of patients [3]. However, even among the initially well-responding CRPC patients, many will develop secondary resistance to ARSIs [3]. Resistance is mainly driven by aberrations of the AR signaling pathway including *AR* gene amplification [4,5], *AR* gene mutations [6,7], and the expression of AR splice variants (e.g., AR variant 7; AR-V7) [8,9,10,11,12]. Although the predictive value of resistance markers such as AR-V7 is controversial [13], liquid biopsy analyses may become important tools to investigate AR alterations directly from blood samples and thereby inform about therapy resistance in real time. Without doubt, early detection of therapy resistance in PC is of utmost importance for patients. Yet, most available liquid biopsy assays are informative only in a subset of patients, as the abundance of circulating biomarkers is generally low, and many assays investigate only one resistance mechanism. We hypothesize that a combination of various liquid biopsy approaches into a more comprehensive multi-analyte approach can provide complementary information and consequently increase the overall sensitivity for tumor-related information.

**Figure 1:**
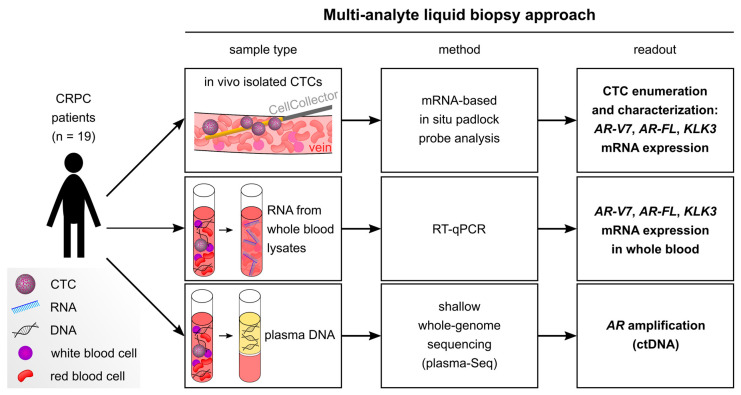
Overview of our multi-analyte liquid biopsy approach which combines enumeration and characterization of in vivo isolated circulating tumor cells (CTCs), mRNA expression analysis in whole blood lysates, and detection of focal *AR* (androgen receptor) amplification in circulating tumor DNA (ctDNA). KLK3: kallikrein-related peptidase 3; CRPC: castration-resistant prostate cancer.

Here, we combined three liquid biopsy approaches to analyze matched CRPC samples. As outlined in Figure 1, we 1) applied the CellCollector for in vivo isolation of circulating tumor cells (CTCs). CTCs were characterized by mRNA in situ padlock probe analysis which visualizes *AR-V7*, *AR* full length (*AR-FL)*, and kallikrein-related peptidase 3 (*KLK3*, often referred to as prostate specific antigen (PSA)) transcripts directly in tumor cells. Furthermore, we 2) developed an RT-qPCR-based assay using a pre-amplification step to assess *AR-V7*, *AR-FL*, and *KLK3* expression in lysed whole blood samples from CRPC patients. Moreover, we 3) investigated *AR* copy-number gain by shallow whole-genome sequencing (sWGS) of plasma DNA. We aimed at identifying which of these liquid biopsy approaches, either single or in combination as a multi-analyte approach, are best suited to obtain tumor-related information.

## 2. Results

### 2.1. Determination of Cut-Off Values for the Identification of CTCs by In Situ Padlock Probe Technology

In situ padlock probe analysis was performed as previously described [14]. The assay was optimized for *AR-V7* detection and could be performed directly on cells attached to the surface of the CellCollector, as well as cells fixed on glass slides. Using this in situ technology, transcripts were visualized within the cells as bright fluorescent spot-like signals which are referred to as rolling circle products (RCPs) [15]. We evaluated the in situ padlock probe assay in cells of four healthy donors captured on the CellCollector, in peripheral blood mononuclear cells (PBMCs) of two healthy donors enriched by density gradient centrifugation of blood samples, as well as in the PC (prostate cancer) cell lines PC-3 (negative control) and VCaP (positive control). As summarized in Table 1, we did not detect any *KLK3* RCPs in the cells of healthy donors or PC-3 cells but identified some cells with one *AR-V7* RCP/cell or one to two *AR-FL* RCPs/cells. These results demonstrate the high specificity of in situ padlock probes, as 99.23–99.29% of PBMCs did not show any false-positive signals for *AR-V7*, and 98.91–99.04% of PBMCs did not show any false-positive signals for *AR-FL*. In VCaP cells, we detected a median of 0 *KLK3* RCPs/cell (interquartile range (IQR) 0–0), 2 *AR-V7* RCPs/cell (IQR 1–4), and 9 *AR-FL* RCPs/cell (IQR 5–13). Although detectable in some VCaP cells, the sensitivity for *KLK3* was very low. Based on the background of *AR-V7* and *AR-FL* observed in healthy controls and PC-3 cells, we defined a cut-off for the identification of CTCs in patient samples. Only cells with ≥1 *KLK3* RCPs/cell, ≥2 *AR-V7* RCPs/cell, or ≥3 *AR-FL* RCPs/cell were rated as CTCs.

### 2.2. In Situ Padlock Probe Technology Allows for the Detection of AR-V7, AR-FL, and KLK3 in CTCs Isolated by CellCollectors

**Figure 2:**
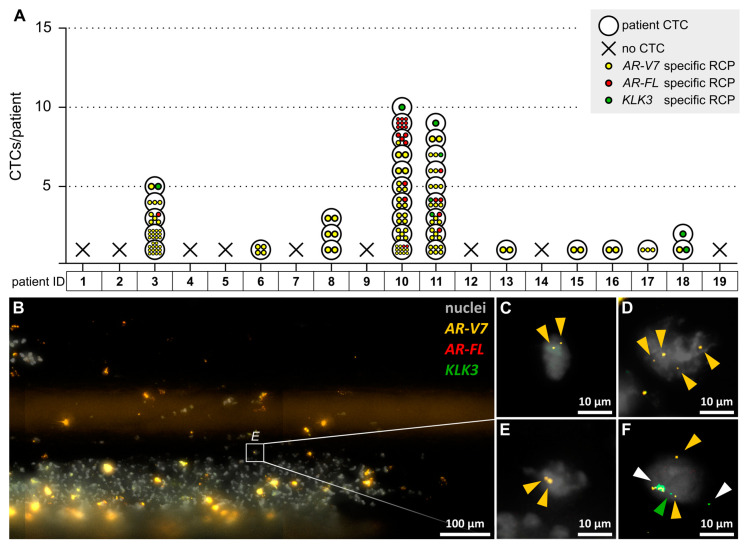
In situ detection of *AR-V7*, *AR-FL*, and *KLK3* transcripts in CTCs of castration-resistant prostate cancer (CRPC) patients. (**A**) For each patient, the number and expression profile of CTCs is visualized. CTCs are represented by black circles. RCPs specific for *AR-V7*, *AR-FL*, and *KLK3* are visualized as yellow, red, and green spots, respectively. Based on the background in healthy controls, only cells with ≥1 *KLK3* RCPs/cell, ≥2 *AR-V7* RCPs/cell or ≥3 *AR-FL* RCPs/cell were rated as CTCs. (**B**) Overview of in-situ-analyzed cells on a CellCollector (patient 16). DAPI-stained nuclei are shown in gray. Scale bar = 100 μm. Representative CTCs from patients 15 (**C**), 6 (**D**), 16 (**E**), and 11 (**F**) are depicted in more detail. Scale bar = 10 μm. *AR-V7-*, *AR-FL-*, and *KLK3*-specific RCPs appear as yellow, red, and green spots, respectively, and are marked by arrowheads. White arrowheads indicate unspecific staining patterns (fluorescent in all channels or signals outside a cell).

We then investigated the CTC status of 19 patients with CRPC. Using the cut-off described above, we identified CTCs in 53% of the patients (10/19), ranging from 1–10 CTCs/patient. Fifty-three percent of the patients (10/19) were positive for *AR-V7-*expressing CTCs. The expression level of *AR-V7* mRNA in CTCs reached up to 14 RCPs/CTC. Sixteen percent of the patients (3/19) were positive for *AR-FL-*expressing CTCs, with expression levels reaching up to 9 RCPs/CTC. Twenty-one percent of the patients (4/19) were positive for *KLK3-*expressing CTCs, with an expression level of 1 RCP/CTC. Most CTCs expressed only one of the investigated transcripts, but some CTCs co-expressed *AR-V7* and *AR-FL*, *AR-V7* and *KLK3*, or all three transcripts. As visualized in Figure 2, intrapatient heterogeneity of CTCs was observed in 21% of the patients (4/19) (patients 3, 10, 11, and 18). In these patients, the expressed transcripts and/or the expression level of transcripts differed between individual CTCs (e.g., a patient having CTCs with low and CTCs with high *AR-V7* expression). Representative images of captured and in-situ-analyzed CTCs are shown in Figure 2. We have already published in situ padlock probe data of CTCs from three patients [14]. Now we have applied a more stringent cut-off for CTC identification and complemented the data with RT-qPCR results.

### 2.3. AR-V7, AR-FL, and KLK3 Expression in Whole Blood Can Be Detected by RT-qPCR

**Figure 3:**
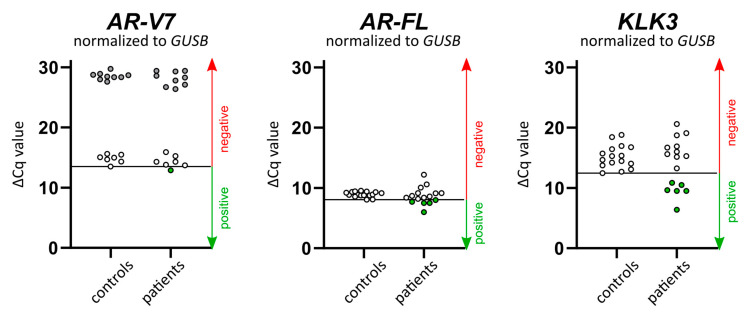
Expression levels of *AR-V7*, *AR-FL*, and *KLK3* in whole blood. Following reverse transcription and preamplification, the expression of *AR-V7*, *AR-FL*, *KLK3*, and β-Glucuronidase (*GUSB*) in whole blood of CRPC patients (*n* = 16) and healthy controls (*n* = 16) was analyzed by qPCR assays. After normalization to *GUSB*, the lowest normalization (ΔCq) value of the control samples was used as the threshold for positive tests, as indicated by the line. Positive tests are depicted as green data points. For *AR-V7*, nine control samples and nine patient samples yielded no Cq-values (45 qPCR cycles). They were assigned a Cq-value of 46, normalized to *GUSB*, and plotted as grey data points.

In addition to the in situ padlock probe analysis, we performed RT-qPCR for the detection of *AR-V7*, *AR-FL*, and *KLK3* transcripts in whole blood of 16 patients using a modified approach from Todenhöfer et al. [16]. We added β-Glucuronidase (*GUSB*) as an internal reference gene for normalization (ΔCq) and used the lowest ΔCq value of negative controls (healthy donor’s blood samples) as a cut-off for positive test results (see also Table S1 and Figure S1 for mean and standard deviation). Six percent of the patients (1/16) were positive for *AR-V7*. Thirty-one percent (5/16) and 38% of the patients (6/16) were positive for *AR-FL* and *KLK3*, respectively (Figure 3). In total, the expression of PC-associated transcripts in whole blood could be detected in 44% of the patients (7/16) using the RT-qPCR assay.

### 2.4. Comparison of Transcript Detection by In Situ CTC Analysis vs Whole Blood RT-qPCR

A subgroup of 16 patients was analyzed by both in situ padlock probe and RT-qPCR, allowing for a direct comparison. More patients were tested positive for *AR-V7* by in situ padlock probe analysis than by RT-qPCR (56% versus 6% of the patients). In contrast, less patients were tested positive for *AR-FL* and *KLK3* by in situ padlock probe analysis than by RT-qPCR (19% versus 31% for *AR-FL* and 25% versus 38% for *KLK3*). The concordance between in situ padlock probe and RT-qPCR assays, i.e., both assays showing the same result, was 38% for *AR-V7* and 63% for *AR-FL* and *KLK3*. Of note, the patient who tested *AR-V7*-positive with the RT-qPCR approach had no detectable *AR-V7*-expressing CTCs. This resistant patient would have been missed, if only the in situ padlock probe analysis had been performed.

### 2.5. AR Focal Amplifications Are Detectable by sWGS

**Figure 4:**
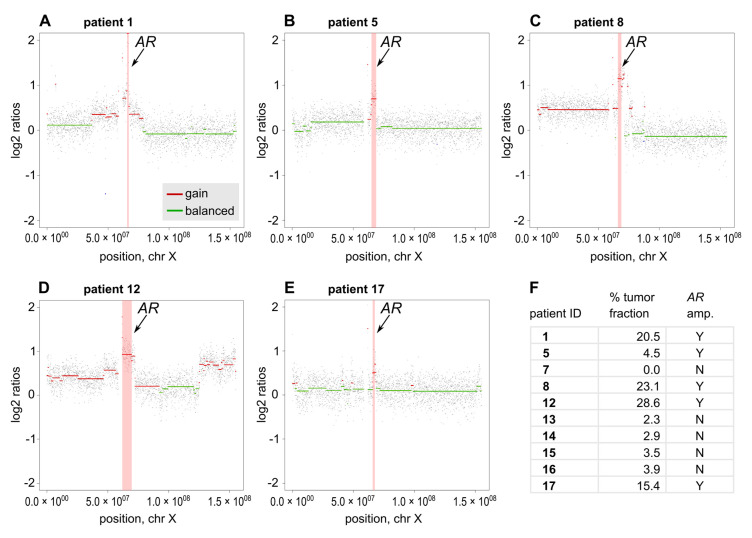
Plasma-Seq reveals copy number alterations and focal *AR* amplification. (**A**–**E**) Log2 ratio plots of the chromosome X of patients with *AR* amplifications (patients 1, 5, 8, 12, and 17). The copy number gain on Xq12, which harbors the *AR* gene, is highlighted. Gains are depicted in red and balanced segments in green. (**F**) Overview of plasma-Seq results of all patients showing % tumor fraction assessed with ichorCNA and presence (Y) or absence (N) of *AR* amplification.

In a subset of ten patients, we had access to corresponding plasma samples, which were analyzed by sWGS, i.e., plasma-Seq [17], to call for focal *AR* amplifications and to quantify tumor content in cell-free DNA using the probabilistic ichorCNA model [18]. Although ichorCNA tumor fractions were below the quantitative threshold of 3% in 30% of the patients (3/10), *AR* amplicons were identified in 50% of the patients (5/10), which is in range with previous findings in advanced PC [7,19]. Representative plots of focal *AR* amplifications are depicted in Figure 4.

### 2.6. A Multi-Analyte Approach Maximizes the Informative Value of Liquid Biopsy Analyses

**Figure 5:**
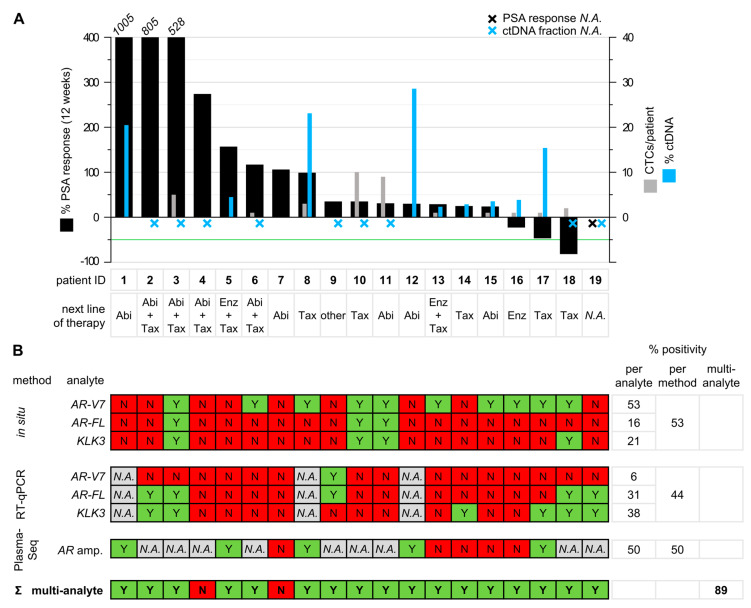
Prostate specific antigen (PSA) response and summary of multi-analyte liquid biopsy approach. (**A**) The waterfall plot depicts best PSA response after 12 weeks (primary y-axis). PSA_50_ response (indicated by the green line) was only reached by one patient. The primary y-axis is cropped at 400%. Values above 400% are printed above the respective bars (patients 1, 2, and 3). The secondary y-axis shows the number of CTCs detected by in situ padlock probe analysis (grey) and the fraction of ctDNA determined by the ichorCNA algorithm (blue). Patient IDs and the next line of therapy are indicated below the bar chart. Abi = abiraterone, Enz = enzalutamide, Tax = taxanes, *N.A.* = not available. (**B**) Summary of positive (Y, green) and negative (N, red) test results of all liquid biopsy analyses for patients 1–19. On the right, the percentage of positive results for each single analyte and the cumulative percentage of positive results across all assays of a given method were calculated. The sensitivity for tumor-related information could be increased by combining all liquid biopsy approaches, resulting in an overall positivity of 89% for the multi-analyte approach (summarized at the bottom). ctDNA: circulating tumor DNA.

The combination of in situ padlock probe analysis of CTCs, RT-qPCR gene expression assays, and plasma-Seq yielded more information than any of the assays alone. In total, PC-associated transcripts could be detected in 53% of the patients (10/19) by in situ padlock probe analysis and in 44% of the patients (7/16) by the RT-qPCR assay. *AR* amplifications could be identified by plasma-Seq in 50% of the patients (5/10). By combining these assays, tumor-related information was obtained in 89% of all patients (17/19). Interestingly, we observed *AR* amplification in circulating tumor DNA (ctDNA) co-occurring with CTC-*AR-V7* expression in two of five patients. In total, we detected the resistance markers *AR-V7* or *AR* amplification in 74% of the patients (14/19). Of note, in 86% of resistant patients (12/14), the resistance was detected by one of the assays exclusively. For example, in patient 5 only *AR* amplification was detected by plasma-Seq, while no *AR-V7* expression was detected by in situ or RT-qPCR analysis. The results of our multi-analyte approach are summarized in Figure 5.

### 2.7. Patient Characteristics and PSA Response

As this study aimed to establish a multi-analyte liquid biopsy approach, we enrolled patients with pretreated progressive-advanced or metastatic CRPC and presumably high levels of ctDNA and CTCs. Specifically, patients had prior treatment with abiraterone (*n* = 4), enzalutamide (*n* = 12), abiraterone and enzalutamide (*n* = 1), or enzalutamide and taxane (*n* = 1). After the sample collection for liquid biopsy analyses, the patients received the next line of therapy including treatment with abiraterone (*n* = 5), enzalutamide (*n* = 1), taxane (*n* = 5), abiraterone and taxane (*n* = 4), enzalutamide and taxane (*n* = 2), or others (*n* = 1). PSA levels in blood were measured at the time of sample collection and monitored for 12 weeks. Three patients showed decreased PSA levels within 12 weeks, but only one of them reached a PSA_50_ response. Fifteen patients showed no PSA response, with PSA levels increasing up to 10-fold from baseline reflecting androgen indifferent disease. For one patient, no data regarding treatment and PSA response was available. Poor PSA response is visualized in the waterfall plot in Figure 5. Median follow-up of patients was 18 months (IQR 10–26 months). At last follow-up, 6 patients had died of disease (31.6%), and 13 were still alive with disease (68.4%). In the univariate linear regression model, none of the identified biomarkers was significantly correlated with change in PSA (all *p* > 0.05; Table S2). Change in PSA was not significantly associated with overall survival (hazard ratio: 1.001; 95% confidence interval: 0.997–1.004; *p* = 0.823). Furthermore, none of the identified biomarkers was significantly associated with altered overall survival (Table S3).

## 3. Discussion

We describe a new multi-analyte assay to detect tumor-related information from liquid biopsies of CRPC patients. This multi-analyte approach includes CTC enumeration and characterization by mRNA-based in situ padlock probe technology, mRNA expression analysis from whole blood lysates by RT-qPCR, and detection of *AR* amplification by plasma-Seq. Our data shows that these liquid biopsy assays complement each other and that their combined use in a multi-analyte approach increases the overall sensitivity for tumor-related information in comparison to the single liquid biopsy assays. We thereby demonstrated the technical feasibility to detect several resistance biomarkers from liquid biopsies, including *AR-V7* transcripts and ctDNA focal *AR* amplifications.

### 3.1. Multi-Analyte Approach Increases Sensitivity

Liquid-biopsy approaches are powerful tools in cancer research although not yet implemented widely in clinical routine. A major limitation remains the low abundance of circulating biomarkers such as ctDNA and CTCs. Therefore, only a subset of patients is positive for ctDNA/CTCs at all, often ranging from 40–70% in metastatic PC disease [5,14,20]. This low “sensitivity” of liquid biopsy assays dramatically hinders their widespread usage in a clinical setting. To overcome this problem, several approaches have been described, such as ultrasensitive sequencing of ctDNA and in-vivo enrichment of CTCs, boosting the sensitivity in general, or including tumor-derived extracellular vesicles as they are more abundant than CTCs [21,22,23,24]. Most importantly, several research articles show that liquid biopsy analytes can complement each other instead of simply being redundant [25,26,27]. This opens the possibility of combining liquid biopsy analytes to increase overall sensitivity for tumor-relevant information, leading to a higher proportion of informative patient blood samples. In our pilot study, we demonstrated that the combinatorial use of analytes enables an 89% detection of tumor-related information in our cohort, suggesting that a multi-analyte liquid biopsy approach improves sensitivity. Similar results were reported for the CancerSEEK approach, where a combination of multiple analytes such as protein and DNA markers improved sensitivity even in early disease ranging from 69–98% depending on tumor entity and stage [28]. In our pilot study, however, the high rate of positivity can also be explained by the advanced CRPC patient cohort. We enrolled patients with high tumor burden and disease progression who had presumably high CTC counts and ctDNA levels. Furthermore, most CRPC patients underwent several lines of therapies, thus, the PSA response of the next line of therapy—irrespective of the administered therapy—remained low as also shown by others [29]. A benefit of our multi-analyte approach is that two distinct resistance mechanisms, i.e., *AR-V7* and *AR* amplification are investigated. Thereby, resistant patients can be identified that may be overlooked if only one liquid biopsy entity was analyzed. This is exemplified in patients 1, 5, and 12 who did not show any *AR-V7* transcripts, neither in CTC nor in whole blood, but *AR* amplification was detected by plasma-Seq.

### 3.2. Background of Cancer-Related Transcripts from Blood Cells Hamper Analysis

An important observation in our multi-analyte approach was the background positivity of PC-related transcripts in the whole blood RT-qPCR approach. All three investigated transcripts, *AR-V7*, *AR-FL*, and *KLK3* showed background signals in blood of healthy donors and thus, we defined a cut-off value based on the healthy donors’ signals [16]. This background decreases the applicability of this method, as only one patient was positive for *AR-V7* based on our cut-off value. As already demonstrated by others, the background signals were possibly derived from blood cells which can also express the investigated transcripts at low levels [30,31,32]. Therefore, the enrichment-free RT-qPCR approach is challenged by a background of blood cell RNA signals. On the other hand, this approach bears the potential to detect transcripts not only from CTCs, but also from exosomes, tumor-derived extracellular vesicles, and cell-free tumor RNA [23,33]. Nevertheless, the major advantage is the inexpensive technical equipment needed so it can easily be performed in parallel to more elaborate liquid biopsy approaches.

Similarly, background positivity for *AR-V7* and *AR-FL* was detected in samples of healthy donors by in situ padlock probe analysis and a cut-off had to be defined to identify CTCs. In a future study, we will further investigate the background from blood cells and include more specific PC markers such as folate hydrolase 1 (*FOLH1*, often referred to as prostate-specific membrane antigen (PSMA)) for a more reliable identification of CTCs. Furthermore, we will use optimized in situ assays for all investigated transcripts [34]. In this study, it has only been optimized for *AR-V7*, which explains the poor detection of *KLK3* in the VCaP cell line and the high positivity of patients for *AR-V7* in comparison to *AR-FL* and *KLK3*. Moreover, we will include the AdnaTest ProstateCancerPanel ARV7 (QIAGEN, Hilden, Germany) as a “gold standard” for *AR-V7* detection [3] in our next study.

### 3.3. In Situ Analysis Reveals Intrapatient Heterogeneity of CTCs

In our pilot study, in situ padlock probe single cell analysis revealed heterogeneous expression patterns of individual CTCs. CTC single-cell analyses are promising tools to monitor clonal evolution and the emergence or disappearance of resistant clones. Furthermore, CTC heterogeneity has been described as poor prognostic marker for response to pathway-specific drugs such as ARSIs. Non-pathway-specific taxane-based chemotherapy on the other hand is less affected by CTC heterogeneity [35]. Therefore, assessment of intrapatient CTC heterogeneity may improve informed treatment decisions. However, the low number of CTCs remains a hurdle to reliably assess CTC heterogeneity. Furthermore, in situ hybridization-based methods are limited to only a few targets, while the feasibility of more comprehensive methods such as single-cell RNA sequencing or in-situ sequencing is hindered by the substantial technical demands and high cost involved [36,37].

### 3.4. AR-Dependent Resistance Mechanisms Can Co-Occur

We investigated two common AR-dependent resistance mechanisms, namely *AR* amplification and *AR-V7* splice variant. Interestingly, we observed that *AR* amplification can co-occur with CTC-*AR-V7* and CTC-*AR-FL* expression. In this context, it is not fully understood how *AR* splicing is regulated. One hypothesis suggests that high availability of *AR* mRNA might be the predominant factor for the generation of *AR-V7* [38,39,40]. Our data suggest that *AR* gain might be a basic mechanism increasing *AR-FL* expression and, hence, the presence of *AR-V7* mRNA. In contrast, a recent study describes dysregulation of splicing-regulatory genes as the driving force for *AR-V7* expression [41] which could explain our observation of *AR-FL*-negative but *AR-V7*-positive CTCs. Furthermore, it is well-documented that both transcripts are co-expressed and that the translated proteins AR-V7 and AR-FL can co-localize and act as agonists inside the cell nucleus [42,43]. Therefore, a combined overexpression of *AR-FL* and *AR-V7* might be a growth advantage for tumor cells. While we observed the co-occurrence of two resistance mechanisms, it was shown by others that *AR* gain and *AR* mutations conferring resistance are described to be inversely correlated [44].

### 3.5. Analysis of AR Mutations and AR-Independent Resistance Mechanisms to Complement Further Studies

AR-V7 has become one of the leading targets of PC research, as various studies suggested that a CTC-based AR-V7 status may serve as a biomarker for treatment selection in CRPC patients [3,11,12,29]. However, the predictive value of AR-V7 is highly controversial and the implementation of AR-V7 tests into clinical routine was discouraged by the panelists of the Advanced Prostate Cancer Consensus Conference 2019 [13]. In addition, only about 25% of CRPC patients test positive for AR-V7, pointing out the importance of alternative resistance mechanisms such as *AR* amplification and *AR* mutations as well as AR-independent resistance mechanisms [12,44]. Our study was limited due to omitting mutation analysis, but this should be included in any future investigation. In addition to *AR* mutations, next-generation sequencing also allows for the detection of AR-independent resistance mechanisms such as treatment-induced neuroendocrine small cell PC [19,45]. Detecting this neuroendocrine PC progression is highly relevant as it requires a different treatment strategy, such as platinum-based chemotherapy [46].

### 3.6. Cohort Size and Availability of Blood Samples

This project was designed as a pilot proof-of-principle study, therefore only a small number of patients were included. Moreover, not all three assays could be performed with all patients due to limited availability of blood samples. Indeed, a limitation of our multi-analyte approach is the need for two different types of blood collection tubes to enable optimal stabilization of RNA (PAXgene Blood RNA tube) and DNA (PAXgene Blood ccfDNA tube), adding up to a total volume of 12.5 mL of blood. Immediately after the collection of blood samples, the CellCollector for in vivo CTC isolation is inserted into the patients’ vein through the intravenous catheter. Thus, no additional blood sample or second venipuncture is needed for CTC analysis. The inclusion of the AdnaTest ProstateCancerPanel ARV7 and ctDNA-mutation analyses, which is planned for our next study, does not necessarily require additional blood samples as several studies have demonstrated the feasibility of ctDNA isolation and analysis from immunomagnetic CTC-depleted blood [47,48].

### 3.7. Correlation of Liquid Biopsy Markers and Patient Outcome

In the present study, we did not discover a potential correlation between liquid biopsy markers and change in PSA or overall survival. This may both be caused by the small case number and the nonstandardized treatment approaches of patients included, with therapeutic regimens individually adapted. Moreover, the current study aimed at improving the diagnostic sensitivity of liquid biopsy approaches for tumor-related information and was not designed to investigate the influence of liquid biopsy markers on patient outcome.

## 4. Materials and Methods

### 4.1. Patient Sampling and Ethics

A total of 19 CRPC patients with high tumor burden and disease progression were enrolled in the study at the Division of Clinical Oncology, Medical University Graz (Austria). The study followed the principles of the World Medical Association Declaration of Helsinki and was approved by the ethical committee (EK28-177-ex-15/16, date: 1 February 2016). A written informed consent was obtained from all patients and healthy donors. Blood samples were collected in PAXgene blood ccfDNA tubes (10 mL blood) (PreAnalytiX, Hombrechtikon, Switzerland), PAXgene blood RNA tubes (2.5 mL blood) (PreAnalytiX), and VACUETTE blood collection tubes K3E K3EDTA (9 mL blood) (Greiner Bio-One, Kremsmünster, Austria). Plasma was extracted from PAXgene blood ccfDNA tubes [49]. PAXgene blood RNA tubes and plasma samples were stored at −80 °C. PBMCs of healthy donors were extracted from VACUETTE blood collection tubes K3E K3EDTA by density gradient centrifugation and cytocentrifuged as previously described with slight modifications [50]. In detail, Lymphoprep density gradient medium (1.077 ± 0.001 g/mL) (Alere Technologies AS, Oslo, Norway) was used. After cytocentrifugation onto glass slides, the PBMCs were fixed with 100% acetone (Merck, Darmstadt, Germany) for 10 min, air-dried for 5 min, and stored at −80 °C until proceeding with in situ padlock probe analysis. CTCs were isolated by in vivo application of the CellCollector CANCER01 detector (GILUPI, Potsdam, Germany). The functionalized tip of the CellCollector was inserted into a patient’s vein through an intravenous catheter as described earlier [51]. After 30 min, the CellCollector was removed, washed 3 times with the post-treatment kit washing solutions 1–3, fixed in 100% acetone for 10 min, and air-dried for 5 min. The CellCollectors were then stored at −80 °C until proceeding with in situ padlock probe analysis. Basic clinical data of the patients was obtained at the time of sample collection and best PSA response was measured within 12 weeks after sample collection.

### 4.2. Cell Lines

The PC cell lines VCaP (kindly provided by Martina Auer, Medical University of Graz, Graz, Austria) and PC-3 (American Type Culture Collection (ATCC), Manassas, VA, USA) were used as positive and negative controls for in situ padlock probe assays. Cell lines were cultured and seeded on glass slides as described previously [14,34]. Seeded slides were fixed in 100% acetone (Merck) for 10 min, air-dried for 5 min, and stored at −80 °C until proceeding with in situ padlock probe analysis.

### 4.3. In Situ Padlock Probe Analysis

In situ padlock probe analysis for the detection of PC-related transcripts *AR-V7*, *AR-FL*, and *KLK3* in PBMCs, VCaP, and PC-3 cells fixed on glass slides and cells captured on the CellCollector was performed as described by El-Heliebi et al. [14]. In short, target-specific reverse transcription primers were used for in situ cDNA synthesis. Padlock probes were hybridized and ligated to the cDNA and amplified by rolling circle amplification. The resulting RCPs were detected by hybridization of fluorescently labelled detection probes. The glass slides and CellCollectors were then scanned on a Zeiss AxioObserver.Z1 fluorescence microscope (Carl Zeiss, Oberkochen, Germany). For the CellCollectors, gaussian smoothing was applied to reduce image noise and in situ signals for *AR-V7*, *AR-FL*, and *KLK3* transcripts were counted manually. In contrast, cells on glass slides were analyzed semi-automatedly using the image analysis software CellProfiler [52]. Signals that were visible in all channels, as well as signals without a direct association to DAPI-stained nuclei, were considered unspecific. A cut-off for the identification of CTCs was set based on the observed background signals in samples of healthy controls. All cells with ≥1 *KLK3* RCPs/cell, ≥2 *AR-V7* RCPs/cell or ≥3 *AR-FL* RCPs/cell were rated as CTCs.

### 4.4. RT-qPCR Gene Expression Assays

In parallel to the in situ approach, we aimed to detect the same tumor-related transcripts in whole blood. For the detection of *AR-V7* and *KLK3* transcripts in whole blood, RT-qPCRgene expression assays were performed [16,53]. Primers for the *AR-FL* assay and TaqMan probes for *AR-V7*, *AR-FL*, and *KLK3* were provided by ViennaLab Diagnostics (Vienna, Austria). To allow for normalization, *GUSB* was included in the gene expression assays as an internal reference [54]. PAXgene blood RNA tube samples were thawed, and RNA was extracted using the PAXgene blood RNA kit (PreAnalytiX). The RNA concentration was measured using the Qubit RNA HS assay kit (Invitrogen, Carlsbad, CA, USA). For reverse transcription, the qPCRBIO cDNA synthesis kit (PCR Biosystems, London, UK) was used. Reverse transcription was run in a total volume of 20 µL with maximal RNA input (400–420 ng or maximal input volume).

Gene-specific preamplification contained SsoAdvanced PreAmp Supermix (Bio Rad, Hercules, CA, USA), primers and TaqMan probes at a final concentration of 50 nM, and 5.00–6.25 µL cDNA in a total volume of 25 µL. Enzyme activation and initial denaturation at 95 °C for 3 min was followed by ten cycles at 95 °C for 15 s (denaturation) and 58 °C for 4 min (primer annealing and elongation), and finally cooling to 4 °C.

qPCR was performed using the RealFast 2x Probe Mix (ViennaLab Diagnostics, Vienna, Austria) primers and TaqMan probes at a final concentration of 250 nM, and 1.8–4.5 µL of preamplified template in a total volume of 20 µL. Enzyme activation at 95 °C for 2 min was followed by 45 cycles of 95 °C for 5 s and 60 °C for 30 s.

Sixteen patient samples and 16 samples of healthy (negative) controls were analyzed by probe-based gene expression assays. Cycle of quantification (Cq) values of *AR-V7*, *AR-FL*, and *KLK3* were normalized to *GUSB*, yielding the respective relative ΔCq values. Technical replicates were averaged (mean) and lowest mean ΔCq value among the negative control samples was set as threshold for positivity as previously described in Todenhöfer et al. [16]. GraphPad Prism version 8.4.1 was used for descriptive statistics and data visualization.

### 4.5. Shallow Whole-Genome Sequencing (sWGS)

To detect *AR* focal amplifications, sWGS, i.e., plasma-Seq, was performed [17]. Briefly, for each patient, 10 mL of blood was collected into PAXgene blood ccfDNA tubes (PreAnalytiX). Cell-free DNA was extracted from 1 mL of plasma using the QIAamp circulating nucleic acid kit (QIAGEN) according to the manufacturer’s protocol. The DNA concentration was measured using the Qubit dsDNA HS assay kit (Thermo Fisher Scientific, Vienna, Austria). Shotgun libraries for plasma-Seq were prepared using the TruSeq Nano DNA sample preparation kit (Illumina, San Diego, CA, USA) according to the manufacturer’s instructions but with three modifications: First, we used 10 ng of input DNA. Second, the fragmentation step was omitted since plasma DNA is known to be highly fragmented. Third, we used 25 PCR cycles for selective amplification of the library fragments [17]. The fragment size distribution and library quality were analyzed using an Agilent DNA 7500 chip on a 2100 Bioanalyzer instrument (Agilent Technologies, Santa Clara, CA, USA). Libraries were quantified by qPCR, pooled equimolarly and sequenced on the Illumina NextSeq platform (Illumina) using the 2 × 75 paired-end mode. On average, 11.31 million reads (range 9.93–12.65 million) were generated per sample. All plasma samples were analyzed with the previously published ichorCNA algorithm to map genome-wide somatic copy number alterations and determine the tumor fraction in plasma DNA [18]. IchorCNA uses a probabilistic model for the simultaneous prediction of large-scale copy number alterations and estimation of tumor fraction in cell-free DNA from ultra-low-pass whole-genome sequencing (ULP-WGS). In parallel, tumor-specific focal events such as focal *AR* amplifications were called as described previously in Ulz et al. [5]. Briefly, reads were mapped to the pseudo autosomal regions (PAR)-masked genome, counted in 50 kb windows and normalized by the total amount of reads. After additional normalization of the GC-content using LOWESS-statistics, resulting read ratios were segmented. From segmented copy number data, we identified focal events using the following criteria: segment should be smaller than 20 Mb; log2 ratio must be higher than 0.2 for amplifications, lower than −0.2 for deletions; segment should contain a gene but not more than 100 genes; log2 ratio must be 0.2 higher than weighted mean of the log2 ratios of neighboring 20 Mb on both sides if it contains a known tumor driver gene or 0.58 higher if it does not contain a known tumor driver gene; neither should segment contain segmental duplications in more than 50% of its size or overlap with known entries in DGVar. Focal identification is done using R and can be accessed online with sample data in IPython notebook format [55].

### 4.6. Statistical Analysis

Statistical analyses of patient data were performed with Stata SE Version 15.1 (StataCorp, College Station, TX, USA). Means and medians were reported with standard deviations (SDs) and IQR, respectively. Change in PSA was calculated taking the best PSA response within 12 weeks, multiplied by 100, divided by the PSA value at baseline. Subsequently, univariate linear regression models were calculated to assess a potential impact of liquid biopsy markers on PSA response. Univariate Cox regression models were calculated to assess an influence of liquid biopsy markers on patient overall survival. Hazard ratios (HR), 95% confidence intervals (95% CI) and *p*-values were provided. A *p*-value of <0.05 was considered statistically significant.

## 5. Conclusions

In our pilot study we assessed a multi-analyte-based approach capable of detecting tumor-related information for its use in a future clinical study. Each liquid biopsy approach has its strengths and limitations summarized as follows: (i) The in situ padlock probe analysis detected *AR-V7* with high sensitivity and in addition allowed for enumeration of CTCs and assessment of intrapatient CTC heterogeneity, thereby providing prognostic and potentially predictive information in 53% of the patients. On the other hand, the in situ padlock probe approach is technically more challenging than the whole-blood RT-qPCR approach and the detection of *AR-V7* is limited to CTCs. (ii) The whole-blood RT-qPCR approach increased the cumulative overall sensitivity of the multi-analyte approach. However, sensitivity for *AR-V7* was low and *AR-FL* and *KLK3* transcripts alone are less relevant for treatment decisions. Therefore, sensitivity needs to be further improved for informative targets such as *AR-V7*. Nevertheless, this whole blood RT-qPCR approach is very attractive due to its low cost, little technical requirements, and the potential to detect tumor RNA not only from CTCs but other sources as well. (iii) Although ctDNA-based plasma-Seq cannot detect *AR-V7* and other mRNA markers, inclusion of the plasma-Seq approach further increased overall sensitivity and provided additional information on therapy resistance. Based on *AR* amplification, which cannot be detected by the mRNA-based approaches, we identified three resistant patients that would have been overlooked otherwise. Moreover, the co-occurrence of two distinct resistance mechanisms that we observed in some patients may be clinically relevant. Overall, the combined multi-analyte liquid biopsy approach showed increased sensitivity, providing tumor-related information in 89% of CRPC patients. We recognize the limitations of our pilot study and will further improve this multi-analyte approach by optimizing our RT-qPCR and in situ padlock probe assays, and by adding mutation analyses and the AdnaTest ProstateCancerPanel ARV7. Moreover, it is crucial to collect specimens at several timepoints longitudinally in a future study to better understand the evolution of resistance mechanisms over time.

## 6. Patents

Amin El-Heliebi, In vivo collection and localized quantification and profiling of circulating cells, proteins and nucleic acids. WO2017081049 A1. Patent pending.

## Figures and Tables

**Table 1:** Cell counts of controls analyzed by in situ padlock probe technology.

**Gene**	**Total Cell Count**	**CellCollector** **Healthy Controls**				**PBMC Healthy Controls**				**PC Cell Lines**			
		**Ctrl 1**	**Ctrl 2**	**Ctrl 3**	**Ctrl 4**	**Ctrl 5**		**Ctrl 6**		**PC-3**		**VCaP**	
		# cells				# cells	%	# cells	%	# cells	%	# cells	%
		N.A.	N.A.	N.A.	N.A.	33,855	100.00	34,544	100.00	4573	100.00	2200	100.00
*KLK3*	0 RCPs/cell	N.A.	N.A.	N.A.	N.A.	33,855	100.00	34,544	100.00	4573	100.00	2181	99.14
	1 RCP/cell	0	0	0	0	0	0.00	0	0.00	0	0.00	18	0.82
	2 RCPs/cell	0	0	0	0	0	0.00	0	0.00	0	0.00	1	0.05
	3 RCPs/cell	0	0	0	0	0	0.00	0	0.00	0	0.00	0	0.00
	>3 RCPs/cell	0	0	0	0	0	0.00	0	0.00	0	0.00	0	0.00
*AR-V7*	0 RCPs/cell	N.A.	N.A.	N.A.	N.A.	33,593	99.23	34,298	99.29	4511	98.64	411	18.68
	1 RCP/cell	5	9	6	6	262	0.77	246	0.71	62	1.36	451	20.50
	2 RCPs/cell	0	0	0	0	0	0.00	0	0.00	0	0.00	423	19.23
	3 RCPs/cell	0	0	0	0	0	0.00	0	0.00	0	0.00	296	13.45
	>3 RCPs/cell	0	0	0	0	0	0.00	0	0.00	0	0.00	619	28.14
*AR-FL*	0 RCPs/cell	N.A.	N.A.	N.A.	N.A.	33,486	98.91	34,213	99.04	4502	98.45	51	2.32
	1 RCP/cell	6	4	1	3	360	1.06	327	0.95	70	1.53	58	2.64
	2 RCPs/cell	0	1	0	0	9	0.03	4	0.01	1	0.02	79	3.59
	3 RCPs/cell	0	0	0	0	0	0.00	0	0.00	0	0.00	121	5.50
	>3 RCPs/cell	0	0	0	0	0	0.00	0	0.00	0	0.00	1891	85.95

This table summarizes the number and percentage of cells with 0, 1, 2, 3, or >3 RCPs/cell for *KLK3*, *AR-V7*, and *AR-FL*. Shaded rows represent the cut-off used for the identification of CTCs in patient samples. Total cell number was not counted on CellCollectors (N.A. = not available). VCaP cells were used as positive control. PBMC: peripheral blood mononuclear cells; RCP: rolling circle product; PC: prostate cancer; Ctrl: control.

## Supplementary Materials

The following are available online at https://www.mdpi.com/2072-6694/12/8/2247/s1, Figure S1: Expression levels of AR-V7, AR-FL, and KLK3 in whole blood, Table S1: Normalized ΔCq values for AR-V7, AR-FL, and KLK3 in lysed whole blood, Table S2: Univariate linear regression model analysing correlation of biomarkers with change in PSA, Table S3: Influence of liquid biopsy markers on overall survival.
