# Supplementary material for: A Multi-Analyte Approach for Improved Sensitivity of Liquid Biopsies in Prostate Cancer
Source: Cancers (Basel). 2020 Aug 11;12(8):2247. doi: 10.3390/cancers12082247 (PMC7465186; doi:10.3390/cancers12082247)
Supplement: Supplementary file 1 [file cancers-12-02247-s001.pdf]

cancers

MDPI

*Article*

# A Multi-Analyte Approach for Improved Sensitivity of Liquid Biopsies in Prostate Cancer

**Lilli Hofmann, Katja Sallinger, Christoph Haudum, Maria Smolle, Ellen Heitzer, Tina Moser, Michael Novy, Kevin Gesson, Thomas Kroneis, Thomas Bauernhofer and Amin El-Heliebi**

## Supplementary Materials

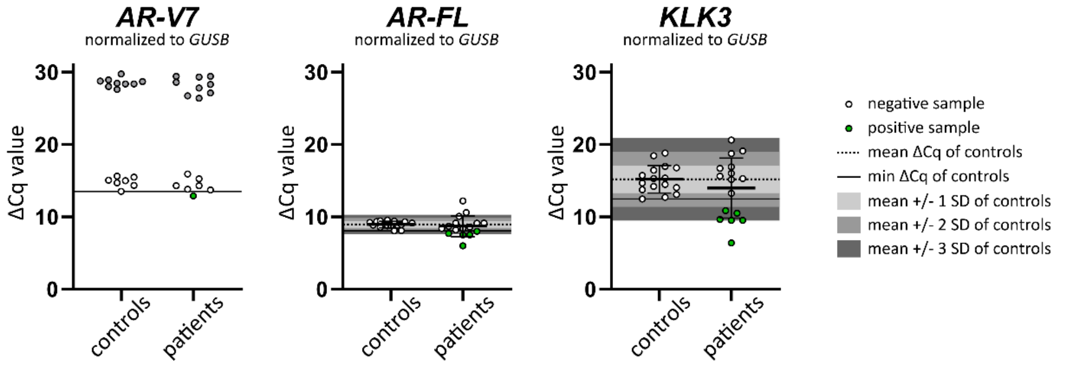


**Figure S1.** Expression levels of *AR-V7*, *AR-FL*, and *KLK3* in whole blood. Following reverse transcription and preamplification, the expression of *AR-V7*, *AR-FL*, *KLK3*, and *GUSB* in whole blood of CRPC patients ($n$ = 16) and healthy controls ($n$ = 16) was analyzed by qPCR assays. Cq values were normalized to *GUSB* (ΔCq). For *AR-V7*, nine control samples and nine patient samples yielded no Cq values (45 qPCR cycles). They were assigned a Cq value of 46, normalized to *GUSB*, and plotted as grey data points. After normalization to *GUSB*, the lowest ΔCq value of the control samples was used as threshold for positive tests, as indicated by the black line. Positive tests are depicted as green data points. For *AR-FL* and *KLK3*, mean and standard deviation (SD) were calculated. The mean of control samples is visualized by the dotted line, and 1, 2, or 3 SD from the mean of the control samples are shaded in grey. Using the lowest ΔCq of the control samples or 2 SD from the mean ΔCq of the control samples as cut-off yielded equal results and no false positives in the control group. However, as mean and SD cannot be calculated for *AR-V7* due to the assigned Cq values of 46, the lowest ΔCq of the control samples was used as cut-off for all transcripts.

**Table S1.** Normalized ΔCq values for *AR-V7*, *AR-FL*, and *KLK3* in lysed whole blood.

| | **ΔCq *AR-V7* Normalized to *GUSB*** | | **ΔCq *AR-FL* Normalized to *GUSB*** | | **ΔCq *KLK3* Normalized to *GUSB*** | |
|---|---|---|---|---|---|---|
| | **Control** | **Patients** | **Control** | **Patients** | **Control** | **Patients** |
| | 15.6 | 12.9 | 8.75 | 6 | 15.7 | 19.1 |
| | 14.35 | 13.8 | 8.85 | 8.4 | 18.45 | 15.3 |
| | 15.05 | 13.7 | 8.85 | 8.2 | 16.75 | 16.9 |
| | 13.5 | 15.9 | 8.75 | 8 | 18.8 | 6.4 |
| | 15.5 | 14.3 | 9.4 | 7.5 | 16.95 | 9.5 |
| | 28.7 | 26.7 | 8.9 | 7.7 | 14.45 | 10.5 |
| | 29.75 | 14.3 | 8.55 | 7.5 | 14.65 | 9.5 |
| | 28 | 27.1 | 8.1 | 9.1 | 13.75 | 16 |
| | 27.6 | 27.8 | 8.8 | 8.4 | 13.1 | 15.6 |
| | 15 | 28.3 | 8.05 | 8.7 | 14.2 | 16.7 |
| | 28.9 | 29.4 | 9.35 | 9.15 | 15.3 | 13.25 |
| | 28.75 | 26.4 | 9.2 | 9.15 | 15.35 | 20.6 |
| | 28.35 | 29.3 | 9.3 | 12.2 | 12.45 | 15.15 |
| | 28.35 | 15.25 | 9.15 | 8.6 | 14.05 | 18.75 |
| | 28.45 | 28.6 | 9.55 | 10.6 | 16.45 | 10.85 |
| | 14.7 | 29.4 | 9.45 | 10.05 | 12.7 | 9.65 |
| mean | N.A. | N.A. | 8.94 | 8.70 | 15.19 | 13.98 |
| 1 SD | N.A. | N.A. | 0.45 | 1.42 | 1.90 | 4.14 |
| 2 SD | N.A. | N.A. | 0.90 | 2.84 | 3.81 | 8.28 |
| 3 SD | N.A. | N.A. | 1.34 | 4.27 | 5.71 | 12.43 |
| mean–1 SD | N.A. | N.A. | 8.49 | 7.28 | 13.29 | 9.84 |
| mean–2 SD | N.A. | N.A. | 8.04 | 5.86 | 11.39 | 5.70 |
| mean–3 SD | N.A. | N.A. | 7.59 | 4.44 | 9.48 | 1.56 |
| min | 13.50 | – | 8.05 | – | 12.45 | – |

ΔCq values of control and patient samples. Several control and patient samples had undetermined Cq values for *AR-V7*. They were set to Cq 46 and normalized to *GUSB* (ΔCq values printed in red). Including these artificial values into the calculation of mean and SD would introduce substantial bias. Likewise, excluding the samples from the calculation would also introduce bias. Therefore, mean and SD were not calculated for *AR-V7* (N.A.–not available). Different cut-offs were applied to identify positive samples and the samples were highlighted accordingly:

| |
|---|
| mean–1 SD of controls |
| mean–2 SD of controls |
| mean–3 SD of controls |

Lowest (min) ΔCq of controls are marked in green. Using the lowest ΔCq of the control samples or 2 SD from the mean ΔCq of the control samples as cut-off yielded the same results and no false positives in the control group. However, as mean and SD cannot be calculated for *AR-V7* due to the assigned Cq values of 46, the lowest ΔCq of the control samples was used as cut-off for all transcripts.

**Table S2.** Univariate linear regression model analysing correlation of biomarkers with change in PSA.

| Change in PSA | Coef. | 95% CI | | SE | *p*-Value |
|---|---|---|---|---|---|
| | | Lower | Upper | | |
| In situ *AR-V7* | −233.44 | −519.37 | 52.49 | 134.88 | 0.103 |
| In situ *AR-FL* | 27.70 | −387.44 | 442.84 | 195.83 | 0.899 |
| In situ *KLK3* | −60.30 | −431.00 | 310.71 | 175.00 | 0.735 |
| RT-qPCR *AR-V7* | −106.64 | −656.23 | 442.96 | 254.40 | 0.682 |
| RT-qPCR *AR-FL* | 255.18 | −16.90 | 527.26 | 125.94 | 0.064 |
| RT-qPCR *KLK3* | 111.19 | −162.55 | 384.92 | 126.71 | 0.396 |
| Plasma-Seq *AR* amp | 157.22 | −314.66 | 629.10 | 204.63 | 0.464 |
| Multi-analyte | −17.36 | −509.88 | 475.16 | 232.33 | 0.941 |
| CTC count | −15.30 | −66.62 | 36.03 | 24.21 | 0.536 |

Legend: Coef.: coefficient; 95% CI: 95% confidence interval; SE: standard error.

**Table S3.** Influence of liquid biopsy markers on overall survival.

| Overall Survival | HR | 95% CI | | *p*-Value |
|---|---|---|---|---|
| | | Lower | Upper | |
| In situ *AR-V7* | 1.185 | 0.205 | 6.839 | 0.850 |
| In situ *AR-FL* | 0.522 | 0.059 | 4.618 | 0.559 |
| In situ *KLK3* | 0.501 | 0.057 | 4.406 | 0.533 |
| RT-qPCR *AR-V7* | 3.517 | 0.361 | 34.213 | 0.279 |
| RT-qPCR *AR-FL* | 2.359 | 0.386 | 14.359 | 0.354 |
| RT-qPCR *KLK3* | 1.325 | 0.220 | 7.986 | 0.759 |
| Plasma-Seq *AR* amp | 3.321 | 0.260 | 42.470 | 0.356 |
| Multi-analyte | 0.698 | 0.081 | 6.000 | 0.743 |
| CTC count | 0.913 | 0.684 | 1.218 | 0.535 |

Legend: HR: hazard ratio; 95% CI: 95% confidence interval.
